# Intelligent Service Chain Orchestration and Resource Allocation in End–Edge Collaborative IIoT Using Multi-Agent Proximal Policy Optimization

**DOI:** 10.3390/s26113583

**Published:** 2026-06-04

**Authors:** Tianzhen Zhao, Bingxin Tian, Lei Wang, Wanming Ma, Bin Wei

**Affiliations:** 1 School of Electronic Engineering, Xidian University, Xi’an 710126, China; 23009100123@stu.xidian.edu.cn; 2 China Mobile Research Institute, Beijing 100053, China; tianbingxin@chinamobile.com (B.T.);

**Keywords:** Industrial Internet of Things (IIoT), Service Function Chain (SFC) orchestration, multi-agent deep reinforcement learning (MADRL), edge computing, resource allocation

## Abstract

The massive heterogeneous data streams and stringent low-latency requirements in the Industrial Internet of Things (IIoT) pose new challenges for edge network resource management. This paper addresses the joint optimization problem of Service Function Chain (SFC) orchestration and resource allocation in edge gateway-assisted IIoT networks, formulated as a mixed-integer nonlinear programming (MINLP) model to minimize end-to-end latency and energy consumption while satisfying quality-of-service (QoS) constraints. To tackle this NP-hard problem and the challenges of partial observability in distributed environments, we propose the SFC Orchestration and Resource Allocation-based Multi-Agent Proximal Policy Optimization (SORA-MAPPO) algorithm. The algorithm adopts a centralized training with decentralized execution (CTDE) paradigm with an intelligent agent cooperation mechanism. Simulation results validate the effectiveness of the proposed scheme in complex IIoT scenarios.

## 1. Introduction

With the advent of fifth-generation (5G) mobile communication technology, the Industrial Internet of Things (IIoT) is driving a profound transformation of manufacturing towards Industry 4.0 [1], whose diverse applications generate massive heterogeneous data streams with stringent low-latency processing requirements [2]. To address the inherent high transmission latency of remote cloud computing [3], mobile edge computing (MEC) offers an effective solution by deploying computing resources at the network edge to meet the demanding latency needs of IIoT applications. However, traditional network architectures still rely on expensive dedicated hardware [4], making it challenging to flexibly adapt to diverse service demands and resulting in high operational costs. Network function virtualization (NFV) decouples network functions from hardware [5], allowing services to be constructed as flexibly orchestratable Service Function Chains (SFCs) composed of virtual network functions (VNFs), significantly enhancing network flexibility and scalability. This SFC-based approach demonstrates broad applicability across various domains: in urban intelligent transportation systems, vehicle-to-infrastructure communications require orchestrated VNF chains for real-time traffic optimization, collision avoidance, and route planning [6]; in smart campus networks, integrated Internet of Things (IoT) sensors and edge gateways coordinate through SFC orchestration to provide seamless connectivity for educational applications, environmental monitoring, and security management [7]; in industrial manufacturing, production line sensors and robotic systems leverage SFC-enabled edge computing for predictive maintenance, quality control, and adaptive manufacturing processes [8]. In such distributed collaborative networks composed of multiple edge gateways that simultaneously host both MEC platforms and VNFs, jointly optimizing VNF orchestration and resource allocation while satisfying quality-of-service (QoS) constraints has become a critical research challenge.

In IIoT environments, computational task offloading to edge gateways is considered a key technology for addressing the limited computing capabilities of resource-constrained terminal devices. Existing research works primarily focus on the joint optimization of task allocation and resource scheduling strategies. Representative studies utilize Lyapunov optimization techniques for delay-aware energy-efficient offloading algorithms [9], design blockchain-enabled computation offloading and resource pricing schemes via Stackelberg game theory [10], and construct comprehensive stochastic computation offloading frameworks and MEC architectures that consider task priority constraints [11,12].

Furthermore, to adapt to highly dynamic network states, recent cutting-edge studies increasingly adopt advanced deep reinforcement learning (DRL) algorithms for intelligent resource management. Specifically, these works propose DRL-based intelligent offloading for blockchain-enabled systems [13], develop personalized federated DRL (PFDRL) for joint offloading and resource allocation in multi-edge environments [14], and design scalable offloading methods combining Proximal Policy Optimization (PPO) with Differentiable Neural Computers (DNCs) to handle complex computation tasks [15]. However, most of these studies overlook the specific requirements and processing order of individual sub-functions within complex services, making their approaches unsuitable for scenarios requiring sophisticated SFC orchestration [16].

SFC orchestration aims to efficiently embed ordered VNF sequences into NFV-enabled IIoT infrastructure while optimizing system performance [17]. Recent works propose online deployment algorithms that optimize latency by prioritizing adjacent paths and considering dynamic network load conditions [18], investigate SFC deployment problems under network function parallelization scenarios by flexibly adjusting parallel VNF resource allocation strategies [19], and study the optimization of SFC deployment and dynamic resource allocation based on VNF performance-resource functions in cloud–edge collaborative environments [20]. However, SFC orchestration is inherently an NP-hard combinatorial optimization problem [21], and traditional optimization methods relied upon by the aforementioned studies face significant computational bottlenecks. Methods based on integer linear programming (ILP) suffer from prohibitively high computational complexity, making it difficult for them to meet real-time requirements in dynamic environments, while heuristic algorithms are prone to getting trapped in local optima with no guarantees of solution quality [22].

Considering the inherent stochasticity of service requests and the dynamic nature of network states in complex IIoT scenarios, DRL has demonstrated great potential for handling such high-dimensional resource management problems due to its online learning capabilities and adaptive decision-making mechanisms [23,24]. Recent works utilize deep Q-networks (DQNs) to achieve distributed SFC embedding in edge computing environments [25], employ natural Actor–Critic algorithms to minimize service latency through joint optimization of SFC routing and wireless resource orchestration among IIoT servers [26], and adopt hierarchical hybrid continuous and discrete action (HHCDA) DRL methods to achieve joint optimization of computational resources and VNF deployment strategies [27]. However, these existing studies often decompose the complex end-to-end SFC deployment problem into a series of independent single-step decisions, thereby ignoring the deep collaborative dilemma caused by partial observability and the intricate sequential dependencies among multi-node VNF deployment decisions in truly distributed environments.

To address the aforementioned challenges and limitations, this paper constructs a unified joint-optimization model in a multi-edge-gateway-assisted IIoT system, where static edge gateways simultaneously serve as both MEC platforms and NFV nodes, aiming to minimize the weighted total cost composed of end-to-end latency and system energy consumption through cooperative optimization of VNF deployment and computational resource allocation. We propose a novel Multi-Agent Proximal Policy Optimization (MAPPO) algorithm based on a multi-agent deep reinforcement learning (MADRL) framework under the centralized training with decentralized execution (CTDE) paradigm, enabling individual agents to effectively utilize global state information to assist learning during the training phase, while requiring only their local observations to make fast and efficient distributed collaborative decisions regarding VNF deployment and computation offloading during the execution phase, thereby achieving optimal responses to dynamic service demands under fixed network topologies.

The main contributions of this article are as follows:

- IIoT services are heterogeneous, spanning ultra-low latency to compute-intensive tasks, which complicates resource offloading and allocation. In an edge gateway–IIoT device cooperative network, SFCs are virtualized into VNFs and offloaded to nodes for flexible orchestration, while scheduling must also address the combined challenges of energy use, end-to-end delay and QoS requirements. A multi-node model is thus established to minimize total system cost by jointly addressing VNF deployment decisions and computation resource allocation, achieving integrated optimization of computation, and deployment.
- To address the challenges of dynamic service demands and large-scale node collaboration in IIoT, this paper proposes the SFC Orchestration and Resource Allocation-based Multi-Agent Proximal Policy Optimization (SORA-MAPPO) algorithm. By integrating SFC orchestration and resource allocation decisions into a unified reinforcement learning framework, SORA-MAPPO uniformly models edge gateways and IIoT devices as independent, cooperative agents and adheres to the CTDE paradigm to learn the optimal policy for complex service processing.
- A multi-edge-gateway-assisted IIoT simulation platform is constructed, and through multi-scenario comparative experiments and hyperparameter tuning, the effectiveness and robustness of SORA-MAPPO in complex environments are validated from multiple dimensions, providing a comprehensive performance evaluation benchmark for SFC orchestration and resource allocation in IIoT scenarios.

## 2. System Model and Problem Formulation

### 2.1. Network Model

**Figure 1:**
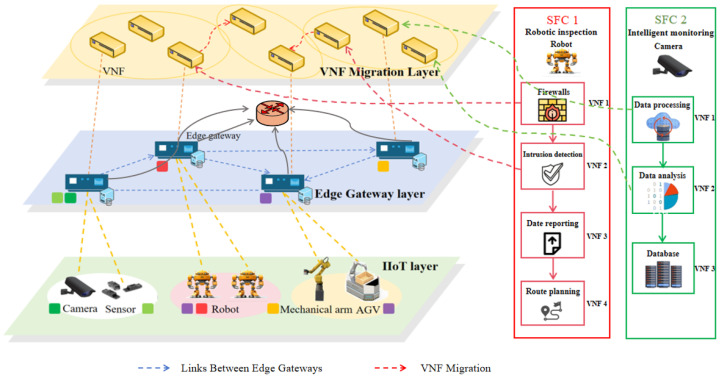
SFC orchestration strategy in multi-edge-gateway-assisted IIoT networks.

As shown in Figure 1, this paper constructs a multi-level distributed computing network for IIoT. This network is mainly composed of the underlying IIoT layer and the upper edge gateway layer. Among these, the IIoT layer includes various terminal devices such as sensors, cameras, robots, and robotic arms, which are responsible for generating diverse business data and computing tasks. Above this, a set of edge gateways constitutes a fixed, distributed edge computing platform, which collaborates with terminal devices through wireless links to complete flexible computing offloading and resource allocation. In order to efficiently meet the differentiated requirements between delay-sensitive services (such as robot patrol) and resource-intensive services (such as intelligent monitoring), this paper abstracts the complex business process into SFCs. As shown in the figure, each SFC consists of a series of ordered VNFs.

**Figure 2:**
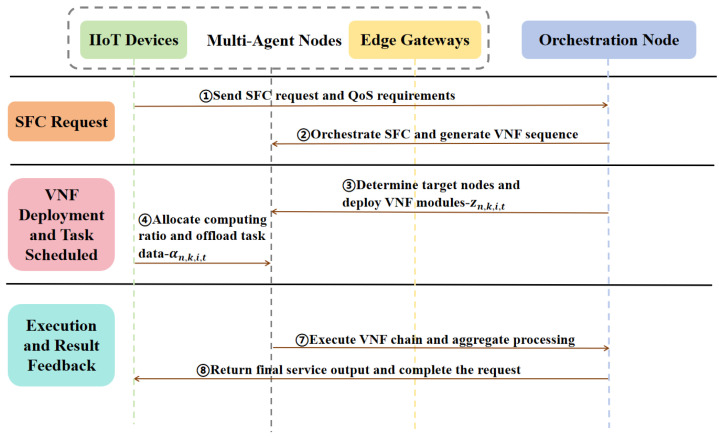
Collaborative multi-agent task flow.

By virtualizing the business functions in the SFC into independent VNF modules, their deployment is no longer restricted by the underlying hardware. This enables each VNF to be used as an independent computing task and be flexibly deployed, instantiated, or migrated to any available node in the network, thus greatly improving the resource utilization efficiency and business processing flexibility of the system. To intuitively illustrate the dynamic interaction process of the proposed network model during SFC request processing, Figure 2 depicts its end-to-end service flow. The process demonstrates how an SFC request is initiated by an IIoT device, collaboratively processed by multiple intelligent nodes through VNF orchestration, deployment, computing allocation, and task offloading, and finally returns the processing results to the requesting IIoT device.

This paper models this hybrid network composed of edge gateways and IIoT devices as a time-varying graph P=(V,E). Here, the vertex set V represents all available computing nodes, which are jointly composed of the IIoT terminal device set D and the edge gateway node set G; that is, V=D∪G. Any computing node i∈V in the network, whether it is an IIoT device d∈D or an edge gateway g∈G, is defined by its inherent computing capacity $C_{i}$, CPU processing rate $F_{i}$, and maximum storage capacity $S_{i}$. The edge set E represents all available wireless communication links between these nodes, and its bandwidth capacity changes according to the relative positions and channel conditions between nodes.

### 2.2. SFC Model

In a dynamic and resource-constrained edge gateway-assisted IIoT environment, the core of service guarantee lies in the efficient orchestration and management of a series of heterogeneous SFC requests. This paper models each SFC request $R_{n}$ as a tuple $R_{n}=\left\{F_{n},C_{n},S_{n},L_{n},\Delta_{n},{\mathrm{type}}_{n},\ldots \right\}$, the core of which is an ordered sequence composed of multiple VNFs, denoted as $F_{n}=\left\{v_{n,1},v_{n,2},\ldots ,v_{n,|F_{n}|}\right\}$. In addition, $C_{n}$ represents the inherent computing complexity of each VNF, that is, the number of CPU cycles required to process each bit of data, denoted as $C_{n}=\left\{c_{n,1},c_{n,2},\ldots ,c_{n,|F_{n}|}\right\}$. Similarly, $S_{n}$ represents the storage resource requirement of each VNF, denoted as $S_{n}=\left\{s_{n,1},s_{n,2},\ldots ,s_{n,|F_{n}|}\right\}$, where $s_{n,k}$ encompasses both the static VNF image footprint and the dynamic state data generated during task execution. $L_{n}=\left\{l_{n,1},l_{n,2},\ldots ,l_{n,|F_{n}|}\right\}$ represents the data packet lengths that each SFC needs to process from IIoT devices, and the length of data packets may change after being processed by a VNF. $l_{n,0}$ and $l_{n,j}$ represent the initial data packet length and the data packet length after being processed by VNF $v_{n,j}$ respectively.

All these complex processing and transmission activities must be completed within the maximum end-to-end delay $\Delta_{n}$ tolerable by the SFC request. To ensure that the constraint of QoS is satisfied, define $T_{n,t}$ as the actual end-to-end delay of the SFC request $R_{n}$ in time slot *t*, which consists of processing delay, transmission delay, VNF instantiation delay, etc. Therefore, this QoS constraint can be expressed as $T_{n,t}\leq \Delta_{n}$. To manage different types of services in a refined manner, the ${\mathrm{type}}_{n}$ attribute divides SFC requests into two categories: one is applications that are extremely sensitive to delay (such as remote real-time control of drones), marked as ${\mathrm{type}}_{n}=1$; the other is computing or data-intensive tasks (such as high-definition video monitoring and analysis), marked as ${\mathrm{type}}_{n}=0$.

In order to optimize the deployment process of the SFC, it is necessary to model it as a set of decision variables. This paper defines the VNF deployment decision variable $z_{n,k,i,t}\in \left\{0,1\right\}$. When $z_{n,k,i,t}=1$, it means that the *k*-th VNF of the SFC request $R_{n}$ is specifically deployed on the network node *i* at the time slot *t*(i∈V). However, relying solely on binary decision variables $z_{n,k,i,t}$ can only determine the deployment of different VNFs in the SFC on different nodes, but it is difficult to accurately describe how multiple nodes jointly share the computing load of the same VNF. Therefore, this paper introduces a computing scheduling ratio variable $\alpha_{n,k,i,t}\in \left[0,1\right]$, which represents the proportion of all computing tasks of VNF $v_{n,k}$ borne by node *i*. The following constraints are satisfied:

(1) $$\begin{array}{cc} \sum_{i\in V}\alpha_{n,k,i,t} & =1\;\forall n,k, \end{array}$$

(2) $$\begin{array}{cc} \alpha_{n,k,i,t} & \leq z_{n,k,i,t}\;\forall n,k,i,t. \end{array}$$

For ease of reference, all notations and variables introduced in the network model and SFC model are consolidated in Table 1.

### 2.3. Communication Model

The communication model is the basis for calculating the end-to-end delay and service cost. In the system of this paper, three key communication scenarios are considered: inter-gateway communication between edge gateways, gateway-to-device communication between edge gateways and IIoT devices, and local communication between IIoT devices.

First, the channel power gain $g_{i,j,t}$ between any two nodes *i* and *j* is defined as

(3) $$g_{i,j,t}=\delta_{i,j}\cdot {\left(d_{i,j,t}\right)}^{-\alpha }\;\forall i,j\in V,i\neq j,$$

where α is the path loss exponent, satisfying α≥2, and $\delta_{i,j}$ represents the small-scale fading of the channel, following the Rayleigh fading model, satisfying $\delta_{i,j}∼\exp\left(\beta \right)$. After establishing the channel model, the data transmission rate $r_{i,j,t}$ is derived according to the Shannon–Hartley theorem:

(4) $$r_{i,j,t}=W_{i,j}\cdot \log_{2}\left(1+\frac{p^{i}\cdot g_{i,j,t}}{N_{0}}\right),$$

where $W_{i,j}$ is the channel bandwidth between nodes *i* and *j*, $p^{i}$ is the transmission power of the transmitting node *i*, and $N_{0}$ is the noise power. Finally, the communication delay and energy consumption generated during the process of the SFC request $R_{n}$ from $v_{n,k-1}$ to $v_{n,k}$ are modeled. First, when the computing tasks of VNF $v_{n,k}$ are distributed across multiple nodes, the scheduling ratio $\alpha_{n,k,i,t}$ fundamentally determines the fraction of the input traffic stream that node *i* is assigned to process. Thus, the actual input data $l_{n,k,i,t}^{\mathrm{in}}$ allocated to node *i* can be expressed as

(5) $$l_{n,k,i,t}^{in}=\alpha_{n,k,i,t}\cdot l_{n,k-1}^{out}.$$

However, not all nodes in the network participate in the calculation of $v_{n,k}$. Therefore, this paper introduces a dynamic set $V_{n,k,t}$ to represent all nodes that actually execute $v_{n,k}$ in time slot *t*, expressed as

(6) $$V_{n,k,t}=\left\{i\in V|z_{n,k,i,t}=1\right\},$$

Based on this set, to ensure data flow conservation, the total input data of VNF $v_{n,k}$ must satisfy the following relation:

(7) $$l_{n,k}^{in}=\sum_{i\in V_{n,k,t}}l_{n,k,i,t}^{in},$$

For notational simplicity, we omit the superscripts in and out in the subsequent formulas. Hereafter, variables indexed with *k* (e.g., $l_{n,k,i,t}$) denote the input data for computing, while variables indexed with k−1 (e.g., $l_{n,k-1}$) denote the output data for transmission. Based on the above definitions, in the inter-gateway link, the communication delay $T_{n,k,t}^{\mathrm{gg}-\mathrm{comm}}$ is expressed as

(8) $$\;T_{n,k,t}^{\mathrm{gg}-\mathrm{comm}}=\;\max_{\begin{array}{c} g\in G_{n,k-1,t}\\ g^{\prime }\in G_{n,k,t} \end{array}}\;\left\{\;\frac{l_{n,k-1,g,t}}{r_{g,g^{\prime },t}}\;\right\},\;$$

where $G_{n,k,t}$ represents the dynamic set of edge gateway nodes that actually execute VNF $v_{n,k}$ at time slot *t*, and the max operator reflects the synchronization requirement arising from distributed computing offloading decisions; as the computational tasks of VNF $v_{n,k-1}$ may be distributed across multiple source nodes for collaborative processing through computing scheduling ratio $\alpha_{n,k-1,i,t}$, these distributed source nodes need to transmit their processing results to multiple destination nodes executing VNF $v_{n,k}$, thus ensuring that VNF $v_{n,k}$ must wait until all data from the slowest transmission link has been received before it can begin processing. Its communication energy consumption $E_{n,k,t}^{\mathrm{gg}-\mathrm{comm}}$ is expressed as

(9) $$\;E_{n,k,t}^{\mathrm{gg}-\mathrm{comm}}=\;\sum_{\begin{array}{c} g\in G_{n,k-1,t}\\ g^{\prime }\in G_{n,k,t} \end{array}}\;p^{g}\;\cdot \;\frac{l_{n,k-1,g,t}}{r_{g,g^{\prime },t}},\;$$

where the summation operator reflects the cumulative nature of energy consumption, as all active transmission links consume energy simultaneously and independently during the communication process, making the total energy consumption the cumulative sum of individual link consumptions. In the gateway-to-device link, the communication delay $T_{n,k,t}^{\mathrm{gd}-\mathrm{comm}}$ is expressed as

(10) $$\begin{array}{c} \;T_{n,k,t}^{\mathrm{gd}-\mathrm{comm}}=\;\max_{\begin{array}{c} g\in G_{n,k-1,t}\\ d\in D_{n,k,t} \end{array}}\;\left\{\;\frac{l_{n,k-1,g,t}}{r_{g,d,t}}\;\right\},\; \end{array}$$

where $D_{n,k,t}$ represents the dynamic set of IIoT device nodes that actually execute VNF $v_{n,k}$ at time slot t. Its total communication energy consumption $E_{n,k,t}^{\mathrm{gd}-\mathrm{comm}}$ is expressed as

(11) $$\begin{array}{c} \;E_{n,k,t}^{\mathrm{gd}-\mathrm{comm}}=\;\sum_{\begin{array}{c} g\in G_{n,k-1,t}\\ d\in D_{n,k,t} \end{array}}\;p^{g}\;\cdot \;\frac{l_{n,k-1,g,t}}{r_{g,d,t}}.\; \end{array}$$

Finally, for the ground link, its communication delay $T_{n,k,t}^{\mathrm{dd}-\mathrm{comm}}$ is expressed as

(12) $$\begin{array}{c} \;T_{n,k,t}^{\mathrm{dd}-\mathrm{comm}}=\;\max_{\begin{array}{c} d\in D_{n,k-1,t}\\ d^{\prime }\in D_{n,k,t} \end{array}}\;\left\{\;\frac{l_{n,k-1,d,t}}{r_{d,d^{\prime },t}}\;\right\},\; \end{array}$$

Its total communication energy consumption $E_{n,k,t}^{\mathrm{dd}-\mathrm{comm}}$ is expressed as

(13) $$\;E_{n,k,t}^{\mathrm{dd}-\mathrm{comm}}=\;\sum_{\begin{array}{c} d\in D_{n,k-1,t}\\ d^{\prime }\in D_{n,k,t} \end{array}}\;p^{d}\;\cdot \;\frac{l_{n,k-1,d,t}}{r_{d,d^{\prime },t}}.\;$$

### 2.4. Computational Model

This paper believes that the total computing delay $T_{n,k,t}^{\mathrm{comp}}$ of a VNF consists of two consecutive stages: the VNF instantiation stage and the data processing stage. Assume VNF $v_{n,k}$ is instantiated in parallel on all selected target nodes. The completion time of the entire instantiation process depends on the slowest node. Therefore, the VNF instantiation delay $T_{n,k,t}^{\mathrm{ins}}$ is defined as

(14) $$T_{n,k,t}^{\mathrm{ins}}=\max_{i\in V}\left\{z_{n,k,i,t}\cdot d_{i,k}^{\mathrm{ins}}\right\},$$

where $d_{i,k}^{\mathrm{ins}}$ is the time required to instantiate VNF $v_{n,k}$ on node *i*. After instantiation, the data processing process is carried out. Since the computing tasks of the VNF are collaboratively assigned to multiple nodes for parallel processing, the total processing delay depends on the slowest node, as the output of $v_{n,k}$ can only be generated after all parallel subtasks have completed their computation. The VNF processing delay $T_{n,k,t}^{\mathrm{proc}}$ is defined as

(15) $$T_{n,k,t}^{\mathrm{proc}}=\max_{i\in V}\left\{z_{n,k,i,t}\;\cdot \;\;\frac{c_{n,k}\;\cdot \;\alpha_{n,k,i,t}\;\cdot \;l_{n,k-1}}{F_{i}}\right\}.$$

The computing energy consumption generated during the processing of the SFC consists of two parts: the energy consumption $E_{n,k,t}^{\mathrm{comp},D}$ generated by the IIoT device and the energy consumption $E_{n,k,t}^{\mathrm{comp},G}$ generated by the edge gateway. The energy consumption calculation is based on the CMOS dynamic power consumption principle, where power consumption is proportional to the square of processor operating frequency, the computational complexity of tasks, and the amount of data processed, which are expressed as

(16) $$\begin{array}{cc} \;E_{n,k,t}^{\mathrm{comp},D} & =\;\;\sum_{d\in D}\;z_{n,k,d,t}\;\cdot \;\kappa_{d}\;\cdot \;c_{n,k}\;\cdot \;l_{n,k,i,t}\;\cdot \;{\left(F_{d}\right)}^{2},\; \end{array}$$

(17) $$\begin{array}{cc} \;E_{n,k,t}^{\mathrm{comp},G} & =\;\;\sum_{g\in G}\;z_{n,k,g,t}\;\cdot \;\kappa_{g}\;\cdot \;c_{n,k}\;\cdot \;l_{n,k,i,t}\;\cdot \;{\left(F_{g}\right)}^{2},\; \end{array}$$

where $\kappa_{i}$ is the effective capacitance coefficient depending on the chip architecture used.

### 2.5. Problem Formulation

This paper aims to conduct a global joint optimization of the edge gateway-assisted edge computing network. The core objective is to co-optimize SFC orchestration and VNF deployment $Z=\{z_{n,k,i,t}\}$ with computing scheduling ratio $A=\{\alpha_{n,k,i,t}\}$ under dynamic service requests and network environments, so as to minimize the total operating cost of the system while ensuring QoS requirements. This total cost is a comprehensive weighted manifestation of the end-to-end service delay and the total system energy consumption. To this end, this paper first defines two key performance indicators of the system: the total delay and the total energy consumption. And we hereby declare two key parameters: $N_{R}$ represents the total number of SFC requests in the current system, while $K_{n}$ represents the length (i.e., the number of VNFs) of the *n*-th SFC request $R_{n}$.

For any SFC request $R_{n}$, its end-to-end total delay $T_{n,t}$ is the accumulation of the communication delay and computing delay of all VNFs on its link:

(18) $$\begin{array}{cc} T_{n,t} & =\sum_{k=1}^{K_{n}}\left(T_{n,k,t}^{\mathrm{comm}}+T_{n,k,t}^{\mathrm{comp}}\right)\\ & =\sum_{k=1}^{K_{n}}\left(T_{n,k,t}^{\mathrm{gg}-\mathrm{comm}}+T_{n,k,t}^{\mathrm{gd}-\mathrm{comm}}+T_{n,k,t}^{\mathrm{dd}-\mathrm{comm}}\right.\\ & \left.+T_{n,k,t}^{\mathrm{ins}}+T_{n,k,t}^{\mathrm{proc}}\right), \end{array}$$

The total energy consumption $E_{t}$ of the system in time slot *t* is the sum of the communication energy consumption and the computing energy consumption generated during the processing of all SFC requests:

(19) $$\begin{array}{cc} E_{t} & =\sum_{n=1}^{N_{R}}\sum_{k=1}^{K_{n}}\left(E_{n,k,t}^{\mathrm{comm}}+E_{n,k,t}^{\mathrm{comp}}\right)\\ \; & =\sum_{n=1}^{N_{R}}\sum_{k=1}^{K_{n}}\left(E_{n,k,t}^{\mathrm{gg}-\mathrm{comm}}+E_{n,k,t}^{\mathrm{gd}-\mathrm{comm}}+E_{n,k,t}^{\mathrm{dd}-\mathrm{comm}}\right.\\ \; & \left.+E_{n,k,t}^{\mathrm{comp},D}+E_{n,k,t}^{\mathrm{comp},G}\right). \end{array}$$

The optimization objectives are as follows:

(20) $$\min_{Z,A}\Phi =w_{T}\sum_{n=1}^{N_{R}}T_{n,t}+w_{E}E_{t},$$

Here, $w_{T}+w_{E}=1$, where $w_{T}$ and $w_{E}$ are adjustable weight factors for balancing latency and energy consumption. Higher $w_{T}$ values prioritize delay optimization for time-sensitive applications (e.g., robotic control), while higher $w_{E}$ values prioritize energy efficiency for battery-powered devices (e.g., remote sensors),

$$\begin{array}{c} \mathrm{subject}\;\mathrm{to} \end{array}$$

(21) $$\begin{array}{cc} C1:\; & \sum_{n=1}^{N_{R}}\sum_{k=1}^{K_{n}}\alpha_{n,k,i,t}\cdot c_{n,k}\cdot l_{n,k-1}\leq C_{i}\;\forall i\in V, \end{array}$$

(22) $$\begin{array}{ccc} & C2:\; & \sum_{n=1}^{N_{R}}\sum_{k=1}^{K_{n}}z_{n,k,i,t}\cdot s_{n,k}\leq S_{i}\;\forall i,t, \end{array}$$

(23) $$\begin{array}{cc} C3:\; & \sum_{i\in V}\alpha_{n,k,i,t}=1\;\forall n,k, \end{array}$$

(24) $$\begin{array}{cc} C4:\; & \alpha_{n,k,i,t}\leq z_{n,k,i,t}\;\forall n,k,i,t, \end{array}$$

(25) $$\begin{array}{ccc} & C5:\; & T_{n,t}\leq \Delta_{n}\;\forall n, \end{array}$$

(26) $$\begin{array}{cc} C6:\; & z_{n,k,i,t}\in \left\{0,1\right\}\;\forall n,k,i, \end{array}$$

(27) $$\begin{array}{cc} C7:\; & \alpha_{n,k,i,t}\in \left[0,1\right]\;\forall n,k,i. \end{array}$$

The optimization objective must be constrained by C1–C7 to ensure its effectiveness. C1 ensures that the computing load allocated to each node does not exceed its maximum computing capacity. C2 guarantees that the cumulative storage requirements of all VNFs deployed on any node, including their image footprint and dynamic state data, do not exceed its physical storage capacity. C3 guarantees that the sum of the proportions of the computing tasks of each VNF allocated on all nodes is 1. C4 ensures that only when a VNF is deployed to a certain node can computing tasks be allocated. C5 ensures that the end-to-end delay of each service chain does not exceed its maximum tolerable delay. C6–C7 constrain the value ranges of the decision variables.

## 3. SORA-MAPPO Algorithm

The joint optimization problem in this paper involves multiple nodes making autonomous yet interdependent decisions on VNF deployment and resource allocation. Since each node can only access local observations, traditional centralized methods face severe challenges in such distributed scenarios. To this end, this paper adopts a MADRL framework, modeling each node as an independent learning agent, and proposes the SORA-MAPPO algorithm based on the CTDE paradigm, where global information guides training while each agent executes decisions independently based on local observations [28].

### 3.1. DEC-POMDP Formulation

The joint optimization problem constructed in this paper is a mixed-integer nonlinear programming (MINLP) problem, which has been proven to be NP-hard. However, traditional optimization methods struggle to effectively address such large-scale and highly dynamic scenarios due to their high computational complexity and reliance on accurate system models. To this end, this paper adopts DRL to solve the problem [29]. DRL, as a data-driven decision-making paradigm, learns through the interaction between agents and the environment to find optimal decisions suitable for such high-dimensional dynamic scenarios. Therefore, this paper models the original optimization problem as a Markov Decision Process (MDP) [30,31], characterized by the tuple 〈S,A,R〉, where S, A, and R represent the state space, action space, and reward function, respectively.

**State Space $S_{i,t}$:** In the multi-agent framework proposed in this paper, all available computing nodes *i* in the network are modeled as agents, and each agent only has partial observation ability to the global environment. At each time slot *t*, the local observation state $S_{i,t}$ of agent *i* consists of three parts: its own state $S_{i,t}^{\mathrm{self}}$, neighborhood state $S_{i,t}^{\mathrm{neighbor}}$, and VNF state $S_{t}^{\mathrm{VNF}}$.

First, the own state $S_{i,t}^{\mathrm{self}}$ is defined as

(28) $$S_{i,t}^{\mathrm{self}}=\left\{f_{i,t},p_{i,t},e_{i,t}\right\},$$

This state reflects the inherent resources and physical constraints of agent *i*, including its remaining computing resources $f_{i,t}$, current location $p_{i,t}$ and remaining energy $e_{i,t}$. Second, the neighborhood state $S_{i,t}^{\mathrm{neighbor}}$ is defined as

(29) $$S_{i,t}^{\mathrm{neighbor}}=\left\{f_{j,t},W_{ij,t}|\forall j\in N_{i,t}\right\},$$

This state describes the interactive environment between the agent and its neighbors within communication range. In this paper, the set of neighborhood nodes of agent *i* at time slot *t* is denoted as $N_{i,t}$, and this set contains all nodes *j* within its communication range. Therefore, it includes the remaining computing resources $f_{j,t}$ of each neighborhood node *j* and the available bandwidth $W_{ij,t}$ of the link connected to agent *i*. Finally, the VNF state $S_{t}^{\mathrm{VNF}}$ is defined as

(30) $$S_{t}^{\mathrm{VNF}}=\left\{c_{n,k},l_{n,k-1},\Delta_{n,t}|\forall v_{n,k}\in V_{t}\right\},$$

This state is constructed as globally shared information reflecting the characteristics of pending service chain requests, and can be observed by each agent *i*. In addition, the set of all VNFs to be processed at time slot *t* is defined as $V_{t}$. Therefore, it includes VNF $v_{n,k}$, the inherent computing complexity $c_{n,k}$, the required data input volume $l_{n,k-1}$, and the remaining delay tolerance $\Delta_{n,t}$ at time slot *t*.

Therefore, the state space $S_{i,t}$ of each agent is represented as

(31) $$S_{i,t}=\left\{S_{i,t}^{\mathrm{self}},S_{i,t}^{\mathrm{neighbor}},S_{t}^{\mathrm{VNF}}\right\}.$$

**Action Space $A_{i}$:** The action $a_{i,t}$ of agent *i* at each time slot *t* is a composite action. This action space $A_{i}$ includes: VNF deployment decision variables $z_{i,t}=\left\{z_{n,k,i,t}|\forall v_{n,k}\in F_{n}\right\}$, used to decide whether the *k*-th VNF $v_{n,k}$ of SFC request $R_{n}$ is deployed on node *i*; and computing resource scheduling ratio variables $\alpha_{i,t}=\left\{\alpha_{n,k,i,t}|\forall v_{n,k}\in F_{n}\right\}$, which allocate computing power to deployed VNFs and finely quantify the proportion of computing power contribution of node *i* to this VNF, thus supporting cross-node collaborative computing. Therefore, the action space of agent *i* at time slot *t* can be expressed as

(32) $$a_{i,t}=\left\{z_{i,t},\alpha_{i,t}\right\}\;a_{i,t}\in A_{i}.$$

To effectively handle this hybrid action space comprising both discrete and continuous variables without artificial discretization, the Actor network of each agent is designed with a multi-head output architecture. Specifically, a Categorical (Softmax) head is utilized to output the discrete VNF deployment decision $z_{n,k,i,t}$, while a parallel Gaussian or Beta distribution head simultaneously generates the continuous resource scheduling ratio $\alpha_{n,k,i,t}$. Both types of actions are generated in a single forward pass, which effectively prevents the precision loss and action-space explosion typically caused by discretization in traditional value-based methods.

**Reward Function $R_{t}$:** The reward function is the core feedback signal provided by the environment to agents, and its design must be consistent with the objective function of the original optimization problem. Therefore, we define the immediate reward $R_{t}$ at each time slot *t* as a composite function consisting of two parts: one part directly reflects the optimization objective, and the other part is a penalty term $P_{t}$ introduced to ensure that key system constraints are satisfied, specifically expressed as

(33) $$R_{t}=-\left(w_{T}\sum_{n=1}^{N_{R}}T_{n,t}+w_{E}E_{t}\right)-P_{t}.$$

The penalty term $P_{t}$ is designed to enforce various system constraints:

(34) $$\begin{array}{cc} P_{t} & =\lambda_{1}\sum_{i\in V}\max\left\{0,\sum_{n=1}^{N_{R}}\sum_{k=1}^{K_{n}}\left(\alpha_{n,k,i,t}\cdot c_{n,k}\cdot l_{n,k-1}\right)-C_{i}\right\}\\ \; & \;+\lambda_{2}\sum_{n=1}^{N_{R}}\sum_{k=1}^{K_{n}}{\left(\sum_{i\in V}\alpha_{n,k,i,t}-1\right)}^{2}\\ \; & \;+\lambda_{3}\sum_{n=1}^{N_{R}}\max\left\{0,T_{n,t}-\Delta_{n}\right\}, \end{array}$$

where $\lambda_{1}$ to $\lambda_{3}$ are penalty coefficients for different constraint violations.In addition to these weighted penalties, the strict logical constraint (C4) is enforced independently via a large constant penalty. This strong negative reinforcement effectively prevents the agent from allocating traffic to non-deployed VNFs.

### 3.2. MAPPO Algorithm Framework

**Figure 3:**
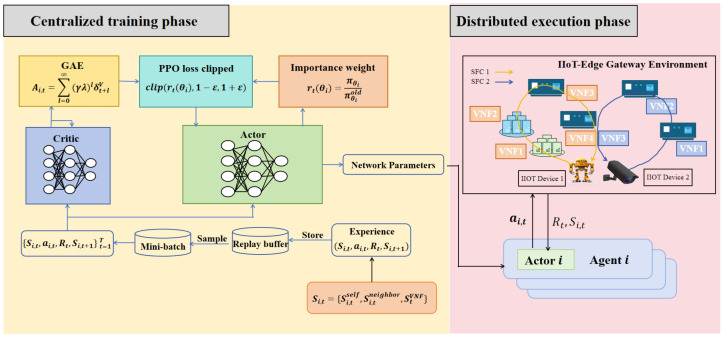
SORA-MAPPO framework.

To learn an optimal action policy in the dynamic environment, this paper constructs a decision-making framework based on SORA-MAPPO. Within this framework, each agent utilizes the observed state vector as a common input for its Actor and Critic networks, thereby enabling environment-aware intelligent decision-making. As shown in Figure 3, the framework adheres to the CTDE paradigm, aiming to achieve efficient collaboration among heterogeneous agents in the edge gateway-assisted IIoT network.

MAPPO is a multi-agent DRL algorithm based on the Actor–Critic framework. In MAPPO, each agent has an independent Actor network, and all agents share a central Critic network during the training phase to evaluate the agents’ actions. The Actor network is defined by parameters $\theta_{i}$, and the Critic network is defined by parameters $\mu_{i}$.The loss function of the Actor network in MAPPO is defined as

(35) $$\;J\left(\;\theta_{i}\;\right)\;=\;E_{t}\;\left[\;\min\;\{\;\varphi_{t}\left(\;\theta_{i}\;\right)\;A_{i,t}\;,\;\;\mathrm{clip}\;\left(\;\varphi_{t}\;\left(\;\theta_{i}\;\right)\;,\;1-\;\varepsilon \;,1+\varepsilon \right)\;A_{i,t}\;\}\right],$$

Here, the function $\mathrm{clip}(r_{t}\left(\theta_{i}\right),1-\varepsilon ,1+\varepsilon )$ restricts $r_{t}\left(\theta_{i}\right)$ within the range of [1−ε,1+ε]. $r_{t}\left(\theta_{i}\right)$ is the importance sampling ratio for training, defined as

(36) $$\varphi_{t}\left(\theta_{i}\right)=\frac{\pi_{\theta_{i}}\left\{a_{i,t}|S_{i,t}\right\}}{\pi_{\theta_{i}}^{\mathrm{old}}\left\{a_{i,t}|S_{i,t}\right\}},$$

Here, $\pi_{\theta_{i}}\left\{a_{i,t}|S_{i,t}\right\}$ is generated by the real-time interaction between the agent and the environment, and $\pi_{\theta_{i}}^{\mathrm{old}}\left\{a_{i,t}|S_{i,t}\right\}$ is the old policy for the interaction between the agent and the environment, which can be sampled from the historical data in the experience pool. Using the importance sampling ratio, the interaction information under the old policy can be used to estimate the new policy, thereby optimizing the new policy and accelerating convergence.

In MAPPO, Generalized Advantage Estimation (GAE) is introduced to estimate the advantage function $A_{i,t}$. The advantage function can effectively reduce the variance of the estimation, and its definition is

(37) $$A_{i,t}=\sum_{l=0}^{\infty }{\left(\gamma \lambda \right)}^{l}\delta_{t+l}^{V},$$

Here, γ represents the discount factor, γ∈[0,1]. λ are the GAE hyperparameters, λ∈[0,1]. $\delta_{t+l}^{V}$ represents the temporal difference error at t+l time and t+l+1 time.

(38) $$\delta_{t+l}^{V}=R_{t+l}+\gamma V_{\mu_{i}}\left(S_{t+l+1}\right)-V_{\mu_{i}}\left(S_{t+l}\right),$$

Here, $V_{\mu_{i}}$ state value function. In addition, the loss function of the Critic network is defined as

(39) $$J\left(\mu_{i}\right)=E_{t}\left[{\left(R_{t}+\gamma V_{\mu_{i}}\left(S_{t+1}\right)-V_{\mu_{i}}\left(S_{t}\right)\right)}^{2}\right].$$

Furthermore, benefiting from the CTDE architecture, SORA-MAPPO achieves zero additional communication overhead during the actual deployment. The information exchange in this framework primarily occurs when agents share observations with the centralized Critic network. However, this centralized training phase is strictly conducted offline on high-performance servers, thus consuming no real-time network bandwidth. During the online application phase, the system operates via fully distributed execution. Agents infer actions locally based on their own observations without any real-time coordination messages. Therefore, the proposed method introduces no extra communication overhead to the live industrial environment, further guaranteeing the ultra-low latency requirements of IIoT services.

### 3.3. Computational Complexity Analysis

Following [32,33], we systematically evaluate the computational complexity of the proposed SORA-MAPPO algorithm. Based on the CTDE paradigm, the total computational overhead primarily depends on the multi-layer perceptron (MLP) architectures of the Actor and Critic networks, as well as the training horizon.

Let $L_{a}$ and $L_{c}$ denote the total number of fully connected (FC) layers in the Actor network and the Critic network, respectively. Similarly, let $u_{j}^{a}$ and $u_{j}^{c}$ represent the number of neurons in the *j*-th layer of the corresponding Actor and Critic networks. Specifically, the output dimension of the Actor network is determined by the hybrid action space, which includes the discrete VNF deployment decision $z_{n,k,i,t}$ and continuous computing scheduling ratio $\alpha_{n,k,i,t}$. For standard FC networks, the computational complexity of a single forward or backward propagation through the *j*-th layer is proportional to the product of the input and output dimensions, i.e., $O(u_{j-1}u_{j})$.

**(1) Complexity in the Training Phase:** During the centralized training phase, as outlined in Algorithm 1, a total of |V| nodes continually interact with the IIoT environment. The training process spans *L* episodes, with each episode containing *T* time steps. In each step, every agent feeds its local observation into the Actor network to generate a policy, while the shared Critic network gathers global states to evaluate the action-value function and compute gradient updates. Consequently, the overall computational complexity during the training phase can be formulated as

(40) $$O\left(\left|V\right|\cdot L\cdot T\cdot \left(\sum_{j=1}^{L_{a}}u_{j-1}^{a}u_{j}^{a}+\sum_{j=1}^{L_{c}}u_{j-1}^{c}u_{j}^{c}\right)\right).$$

**(2) Complexity in the Execution Phase:** Once the training process converges, the system transitions to the decentralized execution phase. In this stage, the centralized Critic network is completely discarded. Each node makes real-time SFC orchestration decisions solely by performing a forward pass through its locally deployed Actor network. Therefore, for a complete operational cycle of *T* steps, the execution complexity for the multi-agent system is drastically reduced to

(41) $$O\left(\left|V\right|\cdot T\cdot \sum_{j=1}^{L_{a}}u_{j-1}^{a}u_{j}^{a}\right).$$

**Algorithm 1** Training phase of SORA-MAPPO  1:Initialize $\theta_{i}$, $\mu_{i}$ and buffer *D*.  2:**for** each episode = 1, 2, …, *L* **do**  3:    **for** each agent *i* **do**  4:        Initialize state $S_{i,1}$ and let $\pi_{\theta_{i}}^{\mathrm{old}}\leftarrow \pi_{\theta_{i}}$  5:    **end for**  6:    **for** each time step *t* = 1, 2, …, *T* **do**  7:        **for** each agent *i* **do**  8:           Obtain $S_{i,t}$ from the environment  9:           Takes action $a_{i,t}$ based on state $S_{i,t}$ according to the policy $\pi_{\theta_{i}}^{\mathrm{old}}$10:           Renew state $S_{i,t+1}$11:       **end for**12:       Obtain a global instant reward $R_{t}$ according to (33)13:       **for** each agent *i* **do**14:           Renew state according to (31)15:           Store ($S_{i,t}$, $a_{i,t}$, $R_{t}$, $S_{i,t+1}$) in buffer *D*16:       **end for**17:       **if** buffer *D* is full **then**18:           Uniformly sample mini-batches from *D*19:           Calculate the GAE ${\left\{A_{i,t}^{t}\right\}}_{t=1}^{T}$ according to (37)20:           Update $\theta_{i}$ by Actor network loss function (35)21:           Update $\mu_{i}$ by Critic network’s loss function (39)22:           Empty *D*23:       **end if**24:     **end for**25:**end for**

## 4. Simulation Results

### 4.1. Simulation Setup

To evaluate the performance of our proposed algorithm, we developed a simulator on a computer running the 64-bit Windows 11 operating system. The machine is equipped with a 13th Gen Intel^®^ Core™ i7-13700H CPU, 16 GB of RAM, and an NVIDIA GeForce RTX 4060 Laptop GPU. All algorithms were implemented in Python 3.9 using the PyTorch 2.5.1 framework. The main configurations are listed in Table 2.

To comprehensively evaluate the performance of our proposed SORA-MAPPO algorithm, we selected four representative algorithms as benchmarks. These algorithms range from fundamental multi-agent reinforcement learning frameworks to classic heuristic methods, aiming to validate the effectiveness and advancement of our algorithm from multiple dimensions.

**(1) MAAC (Multi-Agent Actor–Critic):** An MADRL algorithm that incorporates a soft attention mechanism. In this algorithm, each agent’s critic network can selectively attend to information from other agents based on context, thereby achieving more effective credit assignment. It represents another mainstream approach to MADRL-based collaboration [34].

**(2) DQN (Deep Q-Network):** A typical single-agent DRL algorithm. It combines Q-learning with deep neural networks to handle high-dimensional state spaces, learning the optimal VNF deployment policy through experience replay to maximize long-term rewards.

**(3) PSO (Particle Swarm Optimization):** A classic swarm intelligence algorithm. It simulates bird flock behavior, where particles iteratively update their positions based on personal- and global-best experiences to find a near-optimal VNF deployment strategy [35].

**(4) Greedy:** A classic heuristic algorithm. At each decision point, this algorithm deploys each VNF to the node that minimizes its immediate cost, without considering the impact of decisions on future states and long-term rewards.

To quantitatively assess the performance of the algorithms, we employ the following key metrics:

**(1) Cumulative Reward:** The sum of rewards per episode, directly reflecting the learning efficiency and the quality of the final policy.

**(2) Total End-to-End Delay:** Calculated according to Equation (18) to evaluate the system’s QoS and real-time performance.

**(3) Total System Energy Consumption:** Calculated based on Equation (19) to assess the operational cost and resource efficiency of the system.

**(4) VNF Deployment Rate:** The ratio of successfully deployed VNF instances to total VNF demands. Specifically, a VNF is considered successfully deployed only when it is allocated to a physical node that strictly satisfies both the computing capacity constraint (C1) and the storage capacity constraint (C2). This metric fundamentally measures the system’s resource utilization and deployment capability under multidimensional resource limitations.

**(5) QoS Satisfaction Rate:** The proportion of SFC requests completed within the maximum tolerated delay ($\Delta_{n}$), measuring the system’s ability to guarantee delay commitments.

To ensure statistical reliability, all experiments are evaluated across multiple independent random seeds. Convergence curves display moving averages (solid lines) with variance (shaded regions), while performance comparison points represent averages over multiple trials to eliminate random noise.

### 4.2. Experimental Analysis

**Figure 4:**
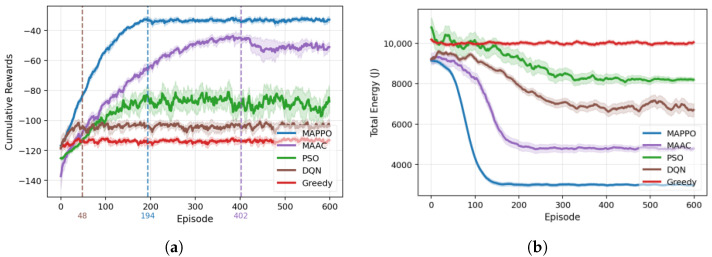
Convergence curves of different algorithms. (**a**) Cumulative reward convergence. (**b**) System energy consumption convergence.

In Figure 4a, SORA-MAPPO demonstrates superior learning efficiency by achieving the highest cumulative reward with the fastest convergence rate. It reaches a stable state at episode 194, approximately 2.07× faster than MAAC, which requires 402 episodes. In terms of practical overhead, SORA-MAPPO reduces the training duration to 8.08 min (compared to 17.11 min for MAAC), benefiting from the PPO clipping and GAE mechanisms that effectively stabilize policy updates. While DQN exhibits the earliest convergence around episode 48, it becomes trapped in a significantly lower reward plateau, reflecting its limited capacity to handle high-dimensional multi-agent coordination. In contrast, the heuristic-based PSO algorithm suffers from significant oscillations and poor stability, struggling to adapt to the highly dynamic IIoT environment. These learning trends are further reflected in the energy consumption curves in Figure 4b. SORA-MAPPO maintains the lowest energy footprint through optimized scheduling, whereas PSO and DQN result in higher overheads due to sub-optimal decision-making. Greedy performs the worst in both metrics due to the lack of long-term strategic planning. Overall, SORA-MAPPO achieves the most robust balance between system utility and energy conservation.

**Figure 5:**
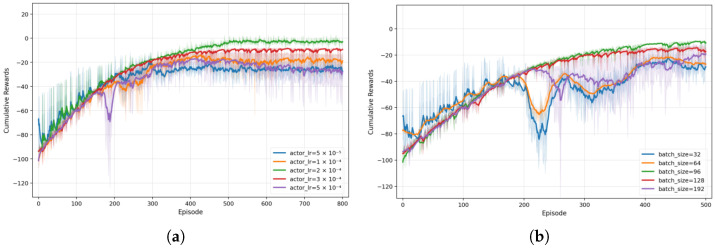
Impact of hyperparameters on training performance. (**a**) Effect of learning rate. (**b**) Effect of batch size.

Figure 5a investigates the impact of the actor learning rate on convergence performance. The results show that a large learning rate of $5\times 10^{-4}$ causes severe gradient oscillations during training and degrades the final performance, while a small learning rate of $5\times 10^{-5}$ converges too slowly and becomes trapped in a local optimum. Therefore, a learning rate of $2\times 10^{-4}$ achieves the best balance between convergence speed and stability, yielding the highest and most robust cumulative rewards. Figure 5b demonstrates the effect of batch size on performance. Small batch sizes (e.g., 32 and 64) lead to severe training instability and massive reward drops due to excessive gradient estimation variance, while an excessively large batch size (e.g., 192) causes training fluctuations and traps the policy in a sub-optimal state. Experiments prove that a batch size of 96 provides the most robust gradient updates, achieving the optimal balance between sample efficiency and stability, thereby guiding the algorithm to learn the most effective policy.

**Figure 6:**
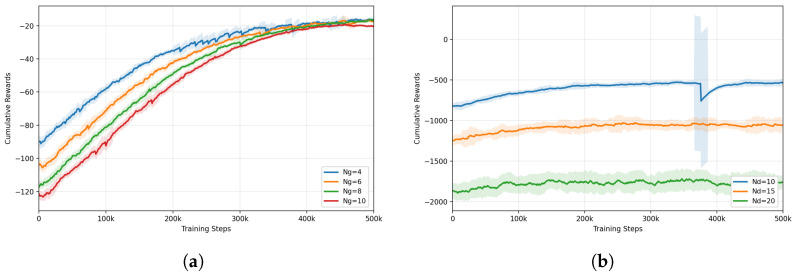
Robustness and scalability analysis of SORA-MAPPO. (**a**) Convergence under varying numbers of edge gateways ($N_{g}$). (**b**) Convergence under varying numbers of IIoT devices ($N_{d}$).

To evaluate the scalability and robustness of SORA-MAPPO, we conducted experiments with varying numbers of edge gateways ($N_{g}$) and IIoT devices ($N_{d}$). Figure 6a shows the convergence as $N_{g}$ increases from 4 to 10. Although a larger $N_{g}$ expands the state–action space and increases initial exploration difficulty, the CTDE architecture enables agents to effectively learn cooperative strategies, eventually converging to a comparable optimal reward level. This demonstrates the algorithm’s excellent scalability in managing distributed edge resources. Figure 6b illustrates system performance under heavier traffic loads, with $N_{d}$ scaling from 10 to 20. More devices generate concurrent SFC requests, intensifying resource competition and lowering the overall reward baseline. Despite this, SORA-MAPPO maintains stable convergence. These results validate the strong robustness and adaptability of the proposed scheme in large-scale IIoT networks.

**Figure 7:**
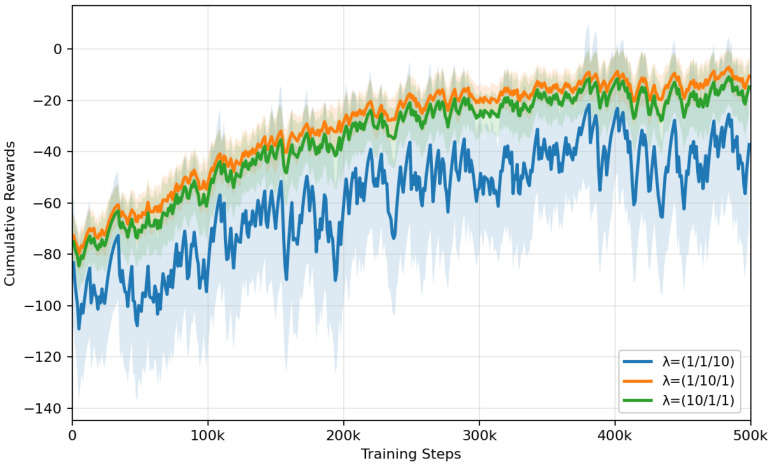
Convergence analysis under different penalty parameter configurations.

Figure 7 illustrates the impact of different penalty coefficient combinations $\left(\lambda_{1},\lambda_{2},\lambda_{3}\right)$ defined in Equation (33) on the convergence performance of the SORA-MAPPO algorithm. Empirical results demonstrate that the configuration $\left(\lambda_{1},\lambda_{2},\lambda_{3}\right)=\left(1,10,1\right)$ yields the highest stable cumulative reward, accompanied by the fastest convergence rate and minimal variance. Conversely, sub-optimal parameter settings induce severe training oscillations and trap the policy optimization in local optima. This instability arises from an imbalanced penalty mechanism that disproportionately penalizes specific constraints, thereby hindering effective environmental exploration. Consequently, the combination (1,10,1) is adopted in our experiments to guarantee a stable and optimal joint orchestration policy.

It is noteworthy that while DQN is evaluated in the convergence analysis, it is excluded from the subsequent performance comparison evaluations. As illustrated in the training results, although SORA-MAPPO efficiently converges to a high reward, DQN fails to learn an effective policy, performing only marginally better than the Greedy approach. The fundamental reason is that our formulated MINLP problem features a massive, hybrid action space (discrete VNF deployment and continuous computational scheduling). DQN, as a value-based method, requires artificial discretization of continuous variables, which triggers a severe “curse of dimensionality.” Consequently, we exclude DQN from performance comparisons to focus on benchmarks that are structurally capable of handling this complexity, such as MAAC and heuristic methods.

**Figure 8:**
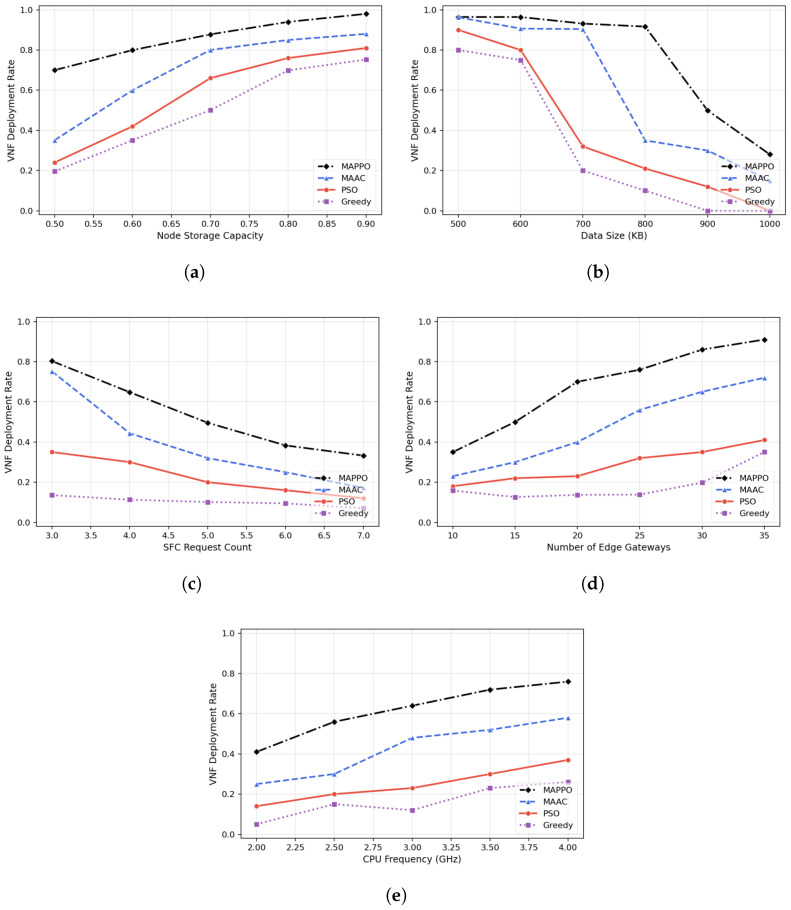
Impact of system parameters on VNF deployment rate. (**a**) Effect of node storage capacity. (**b**) Effect of data size. (**c**) Effect of SFC request count. (**d**) Effect of device number. (**e**) Effect of CPU frequency.

In Figure 8a, increasing node storage capacity improves the deployment rate, as larger storage space allows nodes to accommodate more VNF instances. MAPPO consistently leads because its CTDE framework enables forward-looking planning for the entire SFC chain. In contrast, PSO and Greedy fail to capture complex chain dependencies and only achieve sub-optimal results. In Figure 8b, increasing data size leads to a decline in deployment rates. MAPPO demonstrates the strongest robustness, maintaining a stable rate until 800 KB. However, MAAC’s performance plummets at 800 KB (from 0.9 to 0.35), and Greedy’s rate drops to zero at 900 KB. Although PSO remains non-zero, it stays at an extremely low level, reflecting the inability of these baseline models to handle high transmission overhead. In Figure 8c, more SFC requests intensify resource competition, reducing overall performance. MAPPO achieves efficient load balancing via multi-agent collaboration driven by a shared Critic. Conversely, MAAC and PSO exhibit lower efficiency due to the lack of centralized global evaluation or effective exploration mechanisms. In Figure 8d, MAPPO demonstrates excellent scalability, as more edge gateways provide richer deployment options. MAPPO fully exploits this expansion to optimize VNF placement. In contrast, Greedy and PSO show negligible benefits, proving they cannot efficiently navigate larger solution spaces. In Figure 8e, higher CPU frequency reduces VNF processing latency, enabling more instances to meet delay constraints. MAPPO steadily scales from 0.41 at 2.0 GHz to 0.76 at 4.0 GHz. While MAAC, PSO, and Greedy also show upward trends, their improvements are significantly limited by the structural flaws in their resource-scheduling strategies.

**Figure 9:**
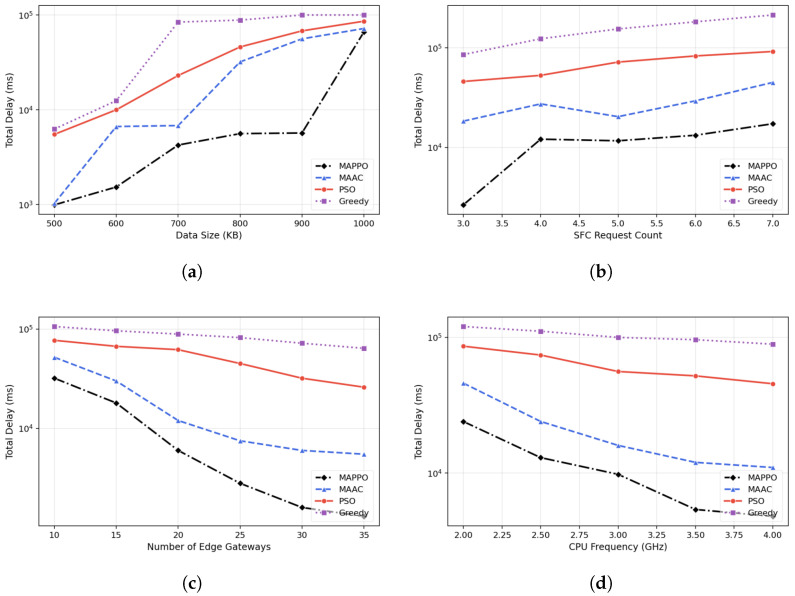
Impact of system parameters on total delay. (**a**) Effect of data size. (**b**) Effect of SFC request count. (**c**) Effect of device number. (**d**) Effect of CPU frequency.

In Figure 9a, increasing data size leads to latency growth for all algorithms. MAPPO maintains the lowest latency across the entire range through joint optimization of VNF deployment and resource allocation. Greedy experiences a sharp latency surge to the order of $10^{5}$ after 600 KB, indicating that its fixed strategy completely fails under high load. MAAC remains relatively stable before 700 KB but deteriorates sharply thereafter. PSO performs better than Greedy but stays significantly higher than MAPPO, as its heuristic search struggles to find optimal solutions in the expanded transmission-intensive space. In Figure 9b, increasing the number of SFC requests causes latency growth due to intensified resource contention. MAPPO’s multi-agent collaboration mechanism enables more intelligent resource allocation, with latency smoothly increasing from approximately 3000 ms at three requests to about 17,000 ms at seven requests. Greedy consistently maintains extremely high latency at the order of $10^{5}$ with almost no variation. MAAC’s latency is consistently about twice that of MAPPO, while PSO fails to effectively coordinate resources across multiple requests, resulting in substantial delays. In Figure 9c, increasing the number of edge gateways reduces system latency, as more gateways distribute computational load and provide shorter transmission paths. MAPPO efficiently explores the expanded decision space, reducing latency from approximately 30,000 ms at 10 gateways to about 2000 ms at 35 gateways, a 93% reduction. Although MAAC and PSO also show latency reduction, PSO’s improvement is limited by its lower exploration efficiency in high-dimensional spaces compared to DRL-based methods. In Figure 9d, higher CPU frequency significantly reduces system latency by accelerating VNF processing. MAPPO’s latency decreases from approximately 25,000 ms at 2.0 GHz to about 5000 ms at 4.0 GHz, an 80% reduction. Greedy maintains extremely high latency at the order of $10^{5}$ with minimal improvement. While MAAC and PSO show moderate reduction, their performance remains substantially inferior to MAPPO, reflecting the structural limitations of their strategies in fully exploiting hardware upgrades.

**Figure 10:**
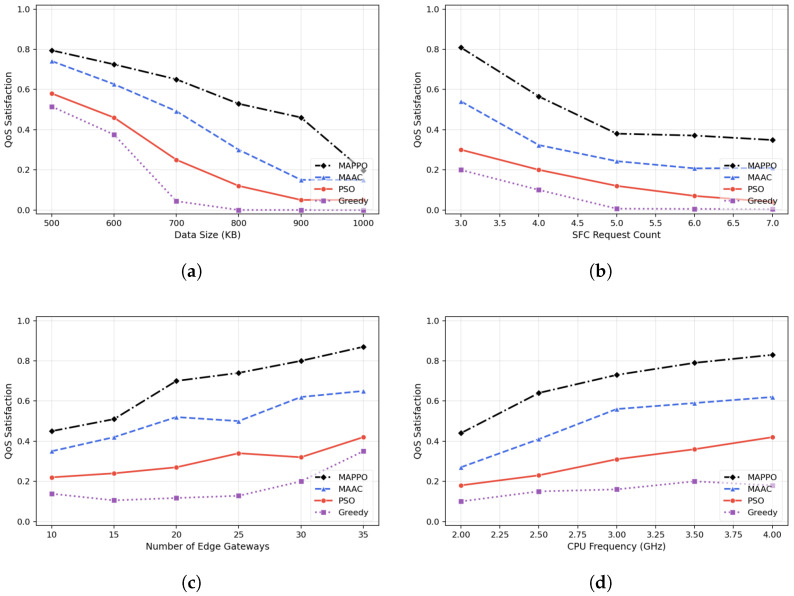
Impact of system parameters on QoS satisfaction. (**a**) Effect of data size. (**b**) Effect of SFC request count. (**c**) Effect of device number. (**d**) Effect of CPU frequency.

In Figure 10a, increasing data size reduces QoS satisfaction due to heavier transmission loads. MAPPO more effectively balances resource demands, declining from 0.8 at 500 KB to approximately 0.45 at 900 KB. Greedy’s QoS collapses to nearly zero at 700 KB, while MAAC and PSO also experience significant degradation, dropping to 0.15 and 0.05 respectively at 900 KB. In Figure 10b, increasing SFC requests reduces QoS due to intensified resource competition. MAPPO’s centralized training enables superior request prioritization, declining from 0.8 at three requests to 0.35 at seven requests while consistently maintaining the highest satisfaction. Greedy drops to 0 after five requests, MAAC remains at approximately half of MAPPO’s level, and PSO degrades rapidly from 0.3 to near zero, indicating structural limitations under severe contention. In Figure 10c, more edge gateways improve QoS by providing additional deployment options. MAPPO quickly adapts to topology changes, improving from 0.45 at 10 gateways to an impressive 0.85 satisfaction at 35 gateways. Greedy shows almost no variation until large-scale deployments. While MAAC and PSO improve, they max out at around 0.65 and 0.42 respectively, falling short of MAPPO due to inadequate exploration of the enlarged solution space. In Figure 10d, higher CPU frequency improves QoS by accelerating VNF processing. MAPPO capitalizes most effectively on enhanced hardware, increasing from 0.44 at 2.0 GHz to 0.84 at 4.0 GHz. Greedy remains largely unresponsive below 0.2, and although MAAC and PSO show moderate improvements reaching 0.62 and 0.42 respectively, their decentralized or heuristic natures prevent them from fully exploiting the augmented resources like MAPPO.

**Figure 11:**
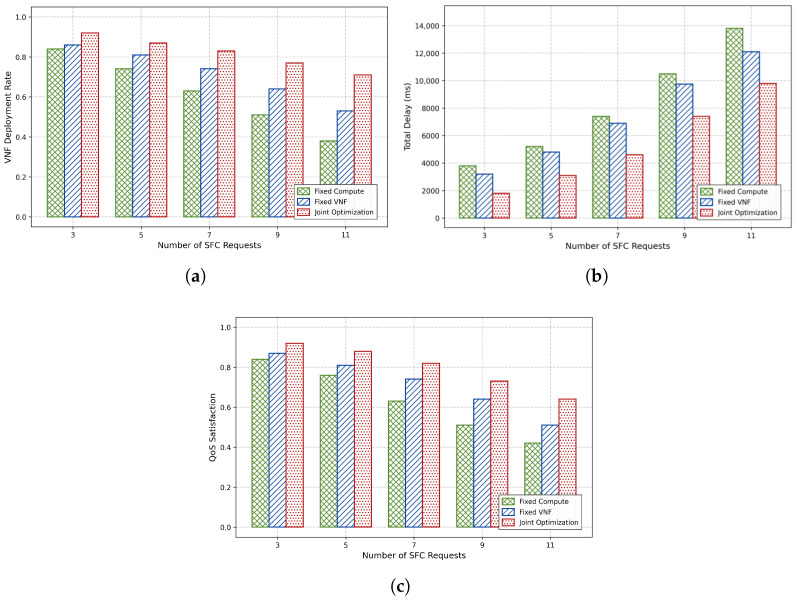
Ablation study of different optimization schemes. (**a**) VNF deployment rate. (**b**) Total delay. (**c**) QoS satisfaction.

To further validate the necessity of the proposed joint-optimization framework, we conducted an ablation study comparing the joint-optimization scheme with two decoupled baselines: Fixed Compute (fixed $\alpha_{n,k,i,t}$) and Fixed VNF (fixed $z_{n,k,i,t}$). As illustrated in Figure 11a, the VNF deployment rate for all schemes declines as the number of SFC requests increases, due to the stringent resource limits of edge nodes. The joint-optimization scheme exhibits the highest deployment capability by dynamically coordinating $z_{n,k,i,t}$ and $\alpha_{n,k,i,t}$ to maximize resource utilization. In contrast, the performance of the Fixed-Compute scheme drops most sharply, as its inability to adjust the computing scheduling ratio leads to frequent violations of the computing capacity constraint C1. Correspondingly, Figure 11b demonstrates that the total delay grows exponentially under heavy traffic loads. According to Equation (15), processing delay is determined by the joint effect of VNF placement and computing allocation. The joint optimization effectively suppresses the acceleration of latency by fine-tuning the load distribution among heterogeneous nodes. Finally, Figure 11c shows the impact on QoS satisfaction. The decoupled schemes lack the flexibility to handle node congestion, easily leading to task timeouts. In contrast, the joint scheme intelligently circumvents congested nodes and allocates sufficient computing resources to urgent tasks, thereby guaranteeing the highest QoS level. These results explicitly prove that the deep coupling of SFC orchestration and resource scheduling is essential. By enabling agents to synergistically evaluate node capacities and network topologies, the joint optimization effectively prevents local resource bottlenecks and latency accumulation.

## 5. Conclusions

To address the challenges posed by massive heterogeneous data streams and stringent low-latency requirements in the Industrial Internet of Things, this paper tackles the joint-optimization problem of Service Function Chain orchestration and resource allocation in edge gateway-assisted IIoT networks. The formulated MINLP model aims to minimize end-to-end latency and system energy consumption through optimizing VNF deployment decisions $z_{n,k,i,t}$ and resource scheduling ratios $\alpha_{n,k,i,t}$ for joint decision-making. The proposed SORA-MAPPO algorithm adopts the CTDE paradigm to model each node as a collaborative agent. Simulation results demonstrate that compared to the MAAC algorithm, the proposed approach achieves improvements of 20%, 42%, and 45% in VNF deployment rate, end-to-end latency, and QoS satisfaction rate, respectively, validating the effectiveness of the proposed scheme.

## Figures and Tables

**Table 1:** Notation and variables in network model and SFC model.

Notation	Description
G	Set of edge gateways, $G=\{1,2,\ldots ,N_{g}\}$
D	Set of IIoT devices, $D=\{1,2,\ldots ,N_{d}\}$
V	Set of all computing nodes, V=G∪D
$N_{g}$	Number of edge gateways
$N_{d}$	Number of IIoT devices
$C_{i}$	Computing capacity of node *i* (CPU cycles per time slot)
$F_{i}$	CPU processing rate of node *i* (cycles/s)
$S_{i}$	Maximum storage capacity of node *i*
$R_{n}$	The *n*-th SFC request
$F_{n}$	Ordered VNF sequence of SFC *n*, $F_{n}=\left\{v_{n,1},v_{n,2},\ldots ,v_{n,|F_{n}|}\right\}$
$v_{n,k}$	The *k*-th VNF of SFC request *n*
$C_{n}$	Computational complexity vector of VNF, $C_{n}=\left\{c_{n,1},c_{n,2},\ldots ,c_{n,|F_{n}|}\right\}$
$c_{n,k}$	Computational complexity of VNF $v_{n,k}$ (CPU cycles/bit)
$S_{n}$	Storage requirement vector of VNF, $S_{n}=\left\{s_{n,1},s_{n,2},\ldots ,s_{n,|F_{n}|}\right\}$
$s_{n,k}$	Storage requirement of VNF $v_{n,k}$ (including image and state data)
$L_{n}$	Data packet length vector, $L_{n}=\left\{l_{n,1},l_{n,2},\ldots ,l_{n,|F_{n}|}\right\}$
$l_{n,0}$	Initial data packet length of SFC request *n* (bits)
$l_{n,k}$	Data packet length after processing by VNF $v_{n,k}$ (bits)
$\Delta_{n}$	Maximum tolerable end-to-end delay of SFC *n* (ms)

**Table 2:** Constant parameters for experiments.

Parameter	Description	Value
$N_{g}$	Number of edge gateways	4
$N_{d}$	Number of IIoT devices	6
$W_{gg}$	Gateway-to-gateway channel bandwidth	20 MHz
$W_{gd}$	Gateway-to-device channel bandwidth	10 MHz
$W_{dd}$	Device-to-device channel bandwidth	5 MHz
$p_{i}$	Transmission power	0.1 W
$N_{0}$	Noise power	$1\times 10^{-13}$ W
$C_{g}$	Gateway computational capacity (per timeslot)	$4.4\times 10^{10}$ cycles
$C_{d}$	Device computational capacity (per timeslot)	$2.2\times 10^{10}$ cycles
$F_{g}$	Gateway CPU processing rate	$3.0\times 10^{9}$ cycles/s
$F_{d}$	Device CPU processing rate	$1.5\times 10^{9}$ cycles/s
$S_{g}$	Gateway maximum storage capacity	50 GB
$S_{d}$	Device maximum storage capacity	8 GB
$\kappa_{g}$	Gateway energy coefficient	$1\times 10^{-27}$
$\kappa_{d}$	Device energy coefficient	$1\times 10^{-28}$
$N_{R}$	Number of SFC requests	3
$|F_{n}|$	VNF sequence length	[3, 4]
$l_{n,0}$	Initial data size	[500,1000] KB
$c_{n,k}$	VNF computational complexity	[1000,5000] cycles/bit
$s_{n,k}$	VNF storage requirement	[100,500] MB
$d_{i,k}^{\mathrm{ins}}$	VNF instantiation time	[0.001, 0.005] s
$\Delta_{n}^{1}$	Max end-to-end delay (delay-sensitive)	[20, 40] s
$\Delta_{n}^{0}$	Max end-to-end delay (computation-intensive)	[40, 60] s

## Data Availability

Data are contained within the article.

## References

[1] Peralta G., Iglesias-Urkia M., Barcelo M., Gomez R., Moran A., Bilbao J. Fog computing based efficient IoT scheme for the Industry 4.0. Proceedings of the 2017 IEEE International Workshop of Electronics, Control, Measurement, Signals and their Application to Mechatronics (ECMSM).

[2] Ning Z., Dong P., Wang X., Hu X., Guo L., Hu B., Guo Y., Qiu T., Leung V.C.M. (2020). When Deep Reinforcement Learning Meets 5G-Enabled Vehicular Networks: A Distributed Offloading Framework for Traffic Big Data. IEEE Trans. Ind. Inform..

[3] Mahmud R., Toosi A.N., Ramamohanarao K., Buyya R. (2020). Context-Aware Placement of Industry 4.0 Applications in Fog Computing Environments. IEEE Trans. Ind. Inform..

[4] Wang S., Chen H., Wang Y. (2022). Collaborative Caching for Energy Optimization in Content-Centric Internet of Things. IEEE Trans. Comput. Soc. Syst..

[5] Xu Y., Zhang T., Liu Y., Yang D., Xiao L., Tao M. (2021). UAV-Assisted MEC Networks with Aerial and Ground Cooperation. IEEE Trans. Wirel. Commun..

[6] Cao H., Lin Z., Yang L., Wang J., Guizani M. (2023). DT-SFC-6G: Digital Twins Assisted Service Function Chains in Softwarized 6G Networks for Emerging V2X. IEEE Netw..

[7] Liu Y., Lu H., Li X., Zhang Y., Xi L., Zhao D. (2021). Dynamic Service Function Chain Orchestration for NFV/MEC-Enabled IoT Networks: A Deep Reinforcement Learning Approach. IEEE Internet Things J..

[8] Asgarian M., Jamshidi K., Bohlooli A. (2024). An Efficient Approximation Algorithm for Service Function Chaining Placement in Edge–Cloud Computing Industrial Internet of Things. IEEE Internet Things J..

[9] Wu H., Chen J., Nguyen T.N., Tang H. (2023). Lyapunov-Guided Delay-Aware Energy Efficient Offloading in IIoT-MEC Systems. IEEE Trans. Ind. Inform..

[10] Lin B., Chen X., Chen X., Ma Y., Xiong N.N. (2024). SGCS: An Intelligent Stackelberg-Game-Based Computation Offloading and Resource Pricing Scheme in Blockchain-Enabled MEC for IIoT. IEEE Internet Things J..

[11] Bebortta S., Senapati D., Panigrahi C.R., Pati B. (2022). Adaptive Performance Modeling Framework for QoS-Aware Offloading in MEC-Based IIoT Systems. IEEE Internet Things J..

[12] Sun L., Wang J., Lin B. (2021). Task Allocation Strategy for MEC-Enabled IIoTs via Bayesian Network Based Evolutionary Computation. IEEE Trans. Ind. Inform..

[13] Chen Z., Yu Z. (2023). Intelligent Offloading in Blockchain-Based Mobile Crowdsensing Using Deep Reinforcement Learning. IEEE Commun. Mag..

[14] Chen Z., Xiong B., Chen X., Min G., Li J. (2024). Joint Computation Offloading and Resource Allocation in Multi-Edge Smart Communities with Personalized Federated Deep Reinforcement Learning. IEEE Trans. Mob. Comput..

[15] Chen Z., Zhang J., Huang Z., Wang P., Yu Z., Miao W. (2024). Computation offloading in blockchain-enabled MCS systems: A scalable deep reinforcement learning approach. Future Gener. Comput. Syst..

[16] Xu S., Li Y., Guo S., Lei C., Liu D., Qiu X. (2022). Cloud–Edge Collaborative SFC Mapping for Industrial IoT Using Deep Reinforcement Learning. IEEE Trans. Ind. Inform..

[17] Song S., Lee C., Cho H., Lim G., Chung J.M. (2020). Clustered Virtualized Network Functions Resource Allocation based on Context-Aware Grouping in 5G Edge Networks. IEEE Trans. Mob. Comput..

[18] Sun G., Xu Z., Yu H., Chang V. (2021). Dynamic Network Function Provisioning to Enable Network in Box for Industrial Applications. IEEE Trans. Ind. Inform..

[19] Agarwal S., Chintapalli V.R., Tamma B.R. FlexSFC: Flexible Resource Allocation and VNF Parallelism for Improved SFC Placement. Proceedings of the 2022 IEEE 8th International Conference on Network Softwarization (NetSoft).

[20] Han Y., Meng W., Fan W. (2024). SFC Placement and Dynamic Resource Allocation Based on VNF Performance-Resource Function and Service Requirement in Cloud-Edge Environment. J. Syst. Eng. Electron..

[21] Guo S., Dai Y., Xu S., Qiu X., Qi F. (2020). Trusted Cloud-Edge Network Resource Management: DRL-Driven Service Function Chain Orchestration for IoT. IEEE Internet Things J..

[22] Quang P.T.A., Hadjadj-Aoul Y., Outtagarts A. (2019). A Deep Reinforcement Learning Approach for VNF Forwarding Graph Embedding. IEEE Trans. Netw. Serv. Manag..

[23] Alsenwi M., Tran N.H., Bennis M., Pandey S.R., Bairagi A.K., Hong C.S. (2021). Intelligent Resource Slicing for eMBB and URLLC Coexistence in 5G and Beyond: A Deep Reinforcement Learning Based Approach. IEEE Trans. Wirel. Commun..

[24] Abedin S.F., Munir M.S., Tran N.H., Han Z., Hong C.S. (2021). Data Freshness and Energy-Efficient UAV Navigation Optimization: A Deep Reinforcement Learning Approach. IEEE Trans. Intell. Transp. Syst..

[25] Chen H., Wang S., Li G., Nie L., Wang X., Ning Z. (2022). Distributed Orchestration of Service Function Chains for Edge Intelligence in the Industrial Internet of Things. IEEE Trans. Ind. Inform..

[26] Li J., Wang R., Wang K. (2023). Service Function Chaining in Industrial Internet of Things with Edge Intelligence: A Natural Actor-Critic Approach. IEEE Trans. Ind. Inform..

[27] Pourghasemian M., Abedi M.R., Hosseini S.S., Mokari N., Javan M.R., Jorswieck E.A. (2023). AI-Based Mobility-Aware Energy Efficient Resource Allocation and Trajectory Design for NFV Enabled Aerial Networks. IEEE Trans. Green Commun. Netw..

[28] Luong N.C., Hoang D.T., Gong S., Niyato D., Wang P., Liang Y., Kim D.I. (2019). Applications of Deep Reinforcement Learning in Communications and Networking: A Survey. IEEE Commun. Surv. Tutor..

[29] Fu X., Yu F.R., Wang J., Qi Q., Liao J. (2019). Service Function Chain Embedding for NFV-Enabled IoT Based on Deep Reinforcement Learning. IEEE Commun. Mag..

[30] Fu X., Yu F.R., Wang J., Qi Q., Liao J. (2020). Dynamic Service Function Chain Embedding for NFV-Enabled IoT: A Deep Reinforcement Learning Approach. IEEE Trans. Wirel. Commun..

[31] Huang H., Zeng C., Zhao Y., Min G., Zhu Y., Miao W., Hu J. (2021). Scalable Orchestration of Service Function Chains in NFV-Enabled Networks: A Federated Reinforcement Learning Approach. IEEE J. Sel. Areas Commun..

[32] Liu W., Li B., Xie W., Dai Y., Fei Z. (2023). Energy Efficient Computation Offloading in Aerial Edge Networks with Multi-Agent Cooperation. IEEE Trans. Wirel. Commun..

[33] Song F., Deng M., Xing H., Liu Y., Ye F., Xiao Z. (2024). Energy-Efficient Trajectory Optimization with Wireless Charging in UAV-Assisted MEC Based on Multi-Objective Reinforcement Learning. IEEE Trans. Mob. Comput..

[34] Chen G., Zhang X., Qi S., Zeng Q., Zhang Y.D. (2024). Network Slicing Resource Allocation Optimization Based on Multiactor-Attention-Critic Joint with Bidding in Heterogeneous Integrated Network. IEEE Syst. J..

[35] Shahab M.H., Sharma Y., Jindal A., Al-Dulaimy A. (2025). A Bi-Objective Policy for Resilient and Sustainable SFC Management in Telco-Cloud Environments. IEEE Access.

